# Functional performance, nutritional status, and body composition in ambulant community-dwelling individuals 1–3 years after suffering from a cerebral infarction or intracerebral bleeding

**DOI:** 10.1186/s12877-016-0226-1

**Published:** 2016-02-19

**Authors:** Birgit Vahlberg, Lena Zetterberg, Birgitta Lindmark, Karin Hellström, Tommy Cederholm

**Affiliations:** Physiotherapy, Department of Neuroscience, Uppsala University, Uppsala, Sweden; Clinical Nutrition and Metabolism, Department of Public Health and Caring Sciences, Uppsala University, Uppsala, Sweden

**Keywords:** Mobility limitations, Stroke, Sarcopenia, Sarcopenic obesity, Physical activity, Obesity, Malnutrition, Ageing

## Abstract

**Background:**

Muscle wasting and obesity may complicate the post-stroke trajectory. We investigated the relationships between nutritional status, body composition, and mobility one to 3 years after stroke.

**Methods:**

Among 279 eligible home-dwelling individuals who had suffered a stroke (except for subarachnoid bleeding) 1–3 years earlier, 134 (74 ± 5 years, 69 % men) were examined according to the Mini Nutritional Assessment-Short Form (MNA-SF, 0–14 points), including body mass index (BMI, kg/m^2^), body composition by bio-impedance analyses (Tanita BC-545), the Short Physical Performance Battery (SPPB, 0–12 points) combining walking speed, balance, and chair stand capacity, and the self-reported Physical Activity Scale for the Elderly (PASE).

**Results:**

BMI ≥ 30 kg/m^2^ was observed in 22 % of cases, and 14 % were at risk for malnutrition according to the MNA-SF. SPPB scores ≤ 8 in 28 % of cases indicated high risk for disability. Mobility based on the SPPB was not associated with the fat-free mass index (FFMI) or fat mass index (FMI). Multivariate logistic regression indicated that low mobility, i.e., SPPB ≤ 8 points, was independently related to risk for malnutrition (OR 4.3, CI 1.7–10.5, *P* = 0.02), low physical activity (PASE) (OR 6.5, CI 2.0–21.2, *P* = 0.02), and high age (OR 0.36, CI 0.15–0.85, *P* = 0.02). Sarcopenia, defined as a reduced FFMI combined with SPPB scores ≤ 8 or reduced gait speed (<1 m/s), was observed in 7 % of cases. None of the individuals displayed sarcopenic obesity (SO), defined as sarcopenia with BMI > 30 kg/m^2^.

**Conclusions:**

Nutritional disorders, i.e., obesity, sarcopenia, or risk for malnutrition, were observed in about one-third of individuals 1 year after stroke. Risk for malnutrition, self-reported physical activity, and age were related to mobility (SPPB), whereas fat-free mass (FFM) and fat mass (FM) were not. Nutrition and exercise treatment could be further evaluated as rehabilitation opportunities after stroke.

## Background

Stroke may affect nutritional status and body composition by causing eating difficulties and reduced mobility [1]. Nutrition and stroke are closely linked. On one hand, obesity and unhealthy eating habits may contribute to stroke events. On the other hand, post-stroke weight loss and malnutrition are common with reports of varying prevalence related to the characteristics of the examined group [2]. In one study of a cohort of older stroke individuals, self-reported weight loss of greater than 3 kg was reported in 26 % 1 year after stroke, and more severe stroke incidents were associated with greater weight loss [3]. Poor food and protein intake may further promote a catabolic state and the progression towards muscle loss and sarcopenia [4]. Conversely, approximately half (52 %) of the individuals in the previously mentioned population-based cohort were overweight at the time of the stroke, and 61 % were overweight after 1 year [3].

Alterations in body composition, such as loss of muscle mass and increased fat mass (FM), are part of physiological ageing. Physical inactivity, poor nutrition, chronic diseases, and hormonal changes may contribute to altered body composition [5]. Sarcopenia is defined as the combination of low muscle mass with low muscle strength and/or function [6]. Individuals who do not regain walking capacity 1 year after stroke display a 6 % loss of leg lean tissue mass [7]. Depending on the diagnostic criteria and the cohort characteristics, the reported prevalence of sarcopenia varies between 1 and 30 % and increases with age in the general population [8]. Hemiparetic stroke in particular may lead to secondary muscle atrophy and specific changes in metabolic and contractile capacity [9].

The combined state of muscle wasting and obesity is known as sarcopenic obesity (SO) [9, 10]. The rates of SO in the elderly are reported to be 2 % and 10 % in those < 70 years and > 80 years, respectively [9]. Although sarcopenia and SO may complicate the post-stroke trajectory [6, 11], data on the prevalence of these conditions after stroke are sparse.

The aims of this study were to determine the physical function and mobility in relation to nutritional status and body composition, i.e., obesity, sarcopenia, and SO in community-dwelling post-stroke individuals one to 3 years after the stroke incident. We hypothesised that mobility in older individuals who had suffered a stroke would be related to fat-free mass (FFM), nutritional status, and physical activity level.

## Methods

### Study design and participants

**Fig. 1:**
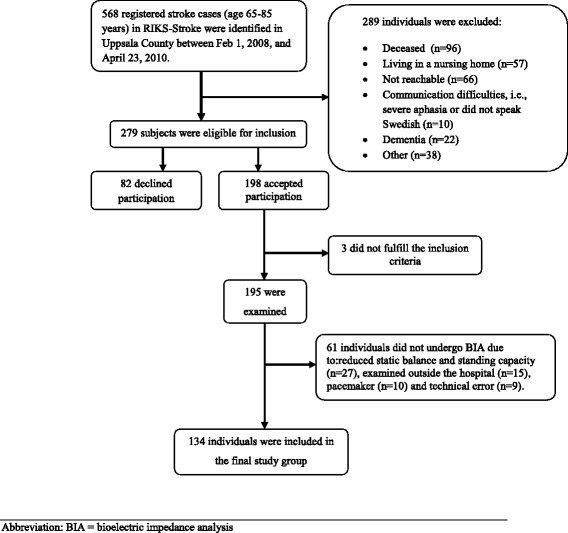
Flow chart describing the inclusion process. Abbreviation: BIA = bioelectric impedance analysis

In this cross-sectional cohort study, eligible individuals were identified by reviewing the national quality discharge register, i.e., the “Riks-Stroke” register, with relevance for Uppsala County, Sweden. Participants were recruited from all stroke patients admitted between February 2008 and April 2010 who were still alive and who had been treated at Uppsala University Hospital during the acute stroke period. Inclusion criteria were as follows: community-dwelling, 65–85 years of age, and a verified stroke (cerebral infarction or intracerebral haemorrhage) within the last one to 3 years. The age span was chosen to cover the period in life when most strokes occur and also to exclude patients for whom age rather than sequelae associated with the stroke may be the strongest contributor to mobility limitations and sarcopenia. A flow chart of the inclusion process is presented in Fig. 1. Exclusion criteria were severe aphasia or cognitive dysfunction with communication problems. Individuals with subarachnoid bleeding are not registered in the national stroke register and were thus not included.

### Procedure

Written study information was sent to the potential community-living participants 1–3 years after the incidence of stroke. After telephone contact, questionnaires on nutrition and physical activity during the previous week were mailed to the participants and completed before the examination. At the scheduled meeting, after signing the written consent for participation, blood samples were drawn. One of the authors (BV) conducted all examinations. The study procedures adhered to the Helsinki Declaration. The study was approved by the Regional Ethical Review Board (Dnr 2009:067) of Uppsala University Hospital, Sweden.

### Data collection

#### Demography, cognition, and morbidity

Age, gender, and co-morbidity data were collected from patient records and questionnaires. The Charlson Co-morbidity Index (CCI) was used to classify co-morbid conditions [12]. Each co-morbid condition was assigned a weight based on severity and was used to generate a patient’s total CCI (0–30 points). The original scale includes cerebrovascular disease and hemiplegia, but these conditions were not included in the CCI for this study [13]. Thus, the CCI had a maximum index score of 27 points, with a higher score indicating more severe co-morbidity. The Short Portable Mental Status Questionnaire (SPMSQ) was used to assess cognition. The SPMSQ consists of 10 questions with a maximum score of 10 points [14]. The scale has been tested for acceptable reliability and validity when administered by telephone [15]. Some data were obtained from the “Riks-Stroke” register at the time of stroke, including cardio-vascular risk factors such as diabetes mellitus, atrial fibrillation, hypertension, smoking, and previous stroke or transient ischaemic attack (TIA). The participants’ need for social support and information on whether they were living alone were gathered from the national stroke register at the 1-year follow-up. Prescribed drugs, including cholesterol-lowering medications, were recorded at the examination.

#### Physical function and activity assessments

Activity limitation was assessed with the Short Physical Performance Battery (SPPB) [16], which generates a scale ranging from 0 to 12 points that includes assessments of balance, gait speed, and chair rising. The SPPB was used to assess performance-based mobility in the lower extremities. An increasing risk of disability has been associated with scores ≤ 8 [6]. To assess walking speed (m/s), the time to perform the 10-m walking test (10 mWT at a self-selected comfortable pace) was recorded using a stop-watch [17]. By converting the 10 mWT (m/s) into 5 subgroups based on walking speed, we were able to give 0 to 4 points for walking speed on the SPPB. Score values were as follows: 0 points (p) = not able to walk, 1 *p* = ≤ 0.43 m/s, 2 *p* = 0.44–0.60 m/s, 3 *p* = 0.61–0.77 m/s, 4 *p* = ≥ 0.78 m/s [18].

Impairments and activity limitation were also assessed with the Modified Motor Assessment Scale, Uppsala University Hospital-99 (M-MAS UAS-99), with a maximum score of 55 indicating good overall performance. This scale is a further development from the original scale by Carr et al. [19]. The M-MAS UAS-99 includes 8 areas of motor function, transfers, arm and hand function, and sitting balance. All of the scales have been used previously in stroke subjects [17, 19, 20] and older individuals [21]. The reliability and validity of all scales have been established [17, 19, 21]. The original MAS was shown to be highly reliable with an inter-rater correlation of 0.95 and an average test-retest correlation of 0.98 [19].

Physical activity was evaluated by the Physical Activity Scale for the Elderly (PASE), which is designed for the self-assessment of physical activity over a 1-week period [22]. The PASE assesses walking, light/moderate or strenuous sports activities, strength training, activities in the household or leisure-time activities, and voluntary work. The scale is scored from 0 upward, with no maximum defined score. The type of activity and the associated frequency and duration were recorded [22]. The PASE has been validated against accelerometer counts and an activity diary and has acceptable test-retest reliability (r = 0.68 to 0.84) and validity (in those above age 70 years, r = 0.64) [23]. Moreover, the PASE has shown moderate correlations with measures of strength, aerobic activity, and balance in individuals with mild stroke [24].

#### Nutritional assessment

The Mini Nutritional Assessment-Short Form (MNA-SF) was used to classify nutritional status [25]. The MNA-SF consists of six items, including BMI, food intake, weight loss, general mobility, acute diseases, psychological stress, and neuropsychological problems [25]. The overall score ranges from 0 to 14 points. Scores of 8–11 points and less than 8 points indicate risk for malnutrition and malnutrition, respectively, whereas scores of 12–14 points indicate normal nutritional status [25]. The MNA-SF is adapted for older populations [26]. The patients’ weights were recorded when they were wearing light indoor clothing. Height was measured to the nearest cm. We used a BMI < 22 kg/m^2^ to indicate underweight because this condition is associated with poor outcomes in patients ≥ 65 years and in individuals who have suffered strokes. A BMI between 22 and 24.9 kg/m^2^ was considered normal weight. A BMI between 25 and 29.9 kg/m^2^ was indicative of being overweight, and values ≥ 30 kg/m^2^ indicated obesity. In addition, an FMI above the 90^th^ percentile of a reference population [27] and an FM% above 30 % for women and 20 % for men were registered [28].

#### Definition of Sarcopenia and Sarcopenic Obesity

Sarcopenia was defined according to the European Working Group on Sarcopenia in Older People [6]. By the use of age- and gender-matched reference data from a group of healthy Swiss adults (2,986 men and 2,649 women) sarcopenia was defined as fat-free mass index (FFMI) below the 25^th^ percentile [27] combined with either an SPPB score ≤ 8 or comfortable gait speed < 1.0 m/s [6]. SO was defined as the combination of sarcopenia (according to the mentioned formula) and BMI > 30 kg/m^2^ [9, 29].

#### Body composition assessments

Bioelectrical impedance analysis (BIA) was performed with a Tanita BC-545 Body Composition Analyzer (Tanita, Inc., Tokyo, Japan). Measurements were performed with the individual in the standing position. The device provides measurements of FFM in kilograms and percentage of total body fat (FM%) according to the equation provided by the software of the machine. The FM index (FMI, kg/m^2^) was calculated as the FM (kg) divided by the height (m) squared. The fat-free mass index (FFMI) (kg/m^2^) was calculated by subtracting the FMI (kg/m^2^) from the BMI (kg/ m^2^). A BIA device (The BC-418 8-contact electrode system (Tanita Corp, Tokyo, Japan) similar to the one used in this study and measuring the subjects in standing position has been validated against dual-energy X-ray absorptiometry (DEXA) and showed good correlations for muscle mass (r = 0.99) and FM (r = 0.87) [30].

#### Biochemical analyses

Non-fasting venous blood samples were obtained, and the biochemical analyses were performed according to the methods of the Department of Clinical Chemistry at the Uppsala Akademiska University Hospital. The samples were frozen at −70 °C until analysis. The biochemical variables included total cholesterol, low-density lipoprotein (LDL) cholesterol, high-density lipoprotein (HDL) cholesterol, plasma albumin, serum insulin-like growth factor-I (IGF-I), and C-reactive protein (CRP). The serum lipid concentrations were considered pathological when the total cholesterol was > 5 mmol/l, LDL cholesterol ≥ 3.0 mmol/l, and HDL cholesterol < 1.2 mmol/l in women and < 1.1 mmol/l in men. The plasma albumin reference value was 34–45 g/l, and the IGF-1 reference value for individuals aged 71–75 years was 64–188 μg/l. A CRP level > 5 mg/l was considered to indicate on-going inflammation.

### Statistical analysis

Descriptive analyses were performed according to the type and distribution of the variables. The differences between men and women and between age classes of 65–74 and 75–85 years were assessed using Student’s t-test for continuous, normally distributed variables and the Mann Whitney U-test for ordinal variables. Categorical variables were compared using Pearson’s chi-squared test (χ^2^), and for non-normal distributed data, Spearman’s rank-order correlation test was used. The significance level was set at *p* < 0.05.

#### Regression analyses

Odds ratios (ORs) and 95 % confidence intervals (CIs) for low versus high mobility, with SPPB as the dependent variable, were calculated to determine independent covariates using multiple logistic regression models. Univariate analyses were conducted to determine significantly associated covariates with mobility (SPPB) and were subsequently tested for multi-collinearity by cross-tabulation. The Statistical Package for the Social Sciences (SPSS), version 23, was used for the analyses (SPSS Inc., Chicago, IL, USA).

## Results

Table 1 presents the baseline characteristics of the 134 participants. A median of 13 months (range: 12 to 38 months) had elapsed between the stroke event and the post-stroke examination.

**Table 1:** Characteristics of the included participants (*n* = 134)

At examination	
Age, years, mean (SD)	74 (7)
Male, n (%)	93 (69)
Living alone, n (%)	35 (26)
Social support, n (%)	
Yes, partial	36 (27)
Yes, complete	11 (8)
At stroke	
Stroke characteristics, n (%)	
Cerebral infarction	117 (87)
Intra-cerebral haemorrhage	17 (13)
Thrombolysis, n (%)	6 (5)
CCI, n (%)	
0	83 (63)
1	37 (27)
2	12 (9)
3	2 (1)
Risk factors for stroke, n (%)	
Earlier stroke	27 (21)
Transient Ischemic Attack	13 (10)
Diabetes	20 (16)
Atrial fibrillation	28 (22)
Hypertension	82 (65)
Smoking	18 (14)
SPMSQ, (0–10 p), median (IQR)	10 (1)

Data were collected from the RIKS-Stroke Register and medical records

*Abbreviations*: *CCI* Charlson comorbidity index based on weighted conditions, 0–27, *SPMSQ* Short Portable Mentale Status Questionnaire

Two-thirds of the participants were men, and all were Caucasian. The gender distribution and age were the same among the eligible subjects who did not participate in the study for various reasons (Fig. 1). Cognitive function (SPMSQ) was high; 71 % of the participants had the highest possible score. Among the 61 participants who could not undergo BIA, the gender distribution (74 % males) and age (75 [5.0] years) were similar, whereas the SPPB score (median of 8 points) was reduced (*p* < 0.001) and the co-morbidity rate was higher (*p* = 0.025) compared with those who underwent BIA.

Data on nutritional status, physical function, body composition, and bio-chemical variables are presented in Table 2. Although MNA-SF indicated a normal nutritional status in general, 14 % were considered at risk for malnutrition. None were malnourished. Overweight was observed in 48 %, and obesity (BMI ≥ 30 kg/m^2^) was observed in 22 % of the subjects.

**Table 2:** Nutritional status body composition, physical function and bio-chemical status, 1–3 years after stroke

		Men		Women		
	Total	65–74 years	75–85 years	65–74 years	75–85 years	Gender difference
	(*n* = 134)	(*n* = 54)	(*n* = 39)	(*n* = 23)	(*n* = 18)	*P*-value
MNA-SF (0–14 p), median (IQR)^a^	14 (2)	14 (1)	13 (2)	13 (2)	13.5 (2)	0.24
BMI (kg/m^2^), mean (SD)	27.3 (4.1)	27.8 (4.0)	26.4 (2.8)	27.4 (4.8)	27 (5.4)	0.68
BMI class, (kg/m^2^), n (%)						
< 22	8 (6)	3 (5.6)	1 (2.6)	2 (8.7)	2 (11.1)	NA
22–24.9	31 (23.3)	11 (20.4)	12 (31.6)	5 (21.7)	3 (16.7)	0.62
25–29.9	64 (48.1)	24 (44.4)	20 (52.6)	12 (52.2)	8 (44.4)	0.17
≥ 30	30 (22.6)	16 (29.6)	5 (13.2)	4 (17.4)	5 (27.8)	0.41
10 mWT (m/s), mean (SD)	1.08 (0.25)	1.16 (0.25)	1.10 (0.22)	0.96 (0.23)	0.94 (0.23)	>0.001
<1.0 m/s, n (%)	38 (28)	11 (12)	7 (7)	11 (27)	9 (2)	
SPPB (0–12 p), median (IQR)	10 (3)	11 (16)	10 (4)	10 (3)	9 (4)	0.005
≤ 8, n (%)	37 (41)	15 (16)	19 (20)	10 (24)	11 (27)	
PASE, (0 ≤ p), mean (SD)	108 (65)	121 (101)	104 (73)	98 (52)	95 (50)	0.18
FFM (kg), mean (SD)						<0.001
Male, mean (SD)	58.5 (6.9)	59.5 (7.4)	56.4 (5.7)			
Female, mean (SD)	41.9 (5.3)			42.4 (4.8)	41.3 (5.9)	
FFMI (kg/m^2^), median (IQR)						<0.001
Male, median (IQR)	19.4 (2.2)	19.5 (2.4)	19.3 (2.3)			
Female, median (IQR)	16.7 (1.5)			16.7 (2.3)	16.7 (2.8)	
FMI (kg/m^2^), median (IQR)						<0.001
Male, median (IQR)	7.3 (3.0)	7.8 (3.2)	7.0 (3.1)			
Female, median (IQR)	10.1 (3.6)			10.1 (3.0)	9.9 (4.6)	
FM%, mean (SD)						<0.001
Male, mean (SD)	27.2 (5.3)	28.0 (5.4)	26.3 (5.2)			
Female, mean (SD)	38.0 (6.3)			37.7 (6.6)	38.4 (6.1)	
Plasma total cholesterol, (mmol/l), mean (SD)	4.9 (1.0)	4.7 (0.94)	4.6 (0.93)	5.4 (1.3)	5.2 (0.93)	0.007
Plasma LDL cholesterol, (mmol/l), mean (SD)	2.5 (0.75)	2.5 (0.78)	2.3 (0.65)	2.6 (0.88)	2.6 (0.69)	0.18
Plasma HDL cholesterol, (mmol/l), mean (SD)	1.4 (0.33)	1.2 (0.25)	1.3 (0.38)	1.5 (0.50)	1.5 (0.29)	<0.001
Plasma albumin (g/l), mean (SD)	38.1 (2.7)	38.6 (2.7)	37.4 (2.5)	38.4 (3.0)	37.2 (2.4)	0.39
Serum IGF-I*, μg/l, mean (SD)	131.1 (46.6)	131.5 (44.9)	134.0 (44.0)	131.8 (56.3)	120.6 (44.7)	0.52
Plasma CRP, mg/l, median (IQR)	1.9 (2.7)	1.9 (2.3)	2.6 (2.3)	1.4 (3.7)	2.3 (2.9)	0.55

Data are means (SD) or medians (IQR). ^a^Reference values for FM%: <20 % (men) or <30 % (women)

*Abbreviations*: *BMI* Body Mass Index, *CRP* C-Reactive Protein, *FM* Fat Mass, *FMI* Fat Mass Index, *FFM* Fat-Free Mass, *FFMI* Fat-Free Mass Index, *HDL* High Density Lipoprotein, *LDL* Low Density Lipoprotein, *MNA-SF* Mini Nutritional Assessment - Short Form, *NA* Non-applicant, *PASE* Physical Activity Scale for the Elderly, *S-IGF-I* Insulin-Like Growth Factor-I, *SPPB* Short Physical Performance Battery, *10 mWT* 10-m Walking Test. *P* < 0.05

^a^For this analysis MNA-SF was calculated with the mobility item omitted

* the IGF-1 reference value for individuals aged 71–75 years was 64–188 μg/l

All participants were able to walk outside according to the mobility item of the MNA-SF questionnaire. A reduced SPPB (≤8 p) was observed in 28 %, with the highest occurrence in old women (Table 2). A low gait speed (<1 m/s) was observed in 28 %, with the highest occurrence in older women (Table 2). FMIs above the 90^th^ percentile of a Swiss reference population [27] were observed in 26 %, and 87 % of the individuals had FM% values above the reference value. FFMI was low (defined as < 25^th^ percentile of a Swiss reference population) in 14 % of the individuals [27]. Sarcopenia, i.e., the combined finding of reduced FFMI with either low SPPB (≤8 points) or low gait speed (<1.0 m/s), was observed in 7 %. None of these sarcopenic individuals were also obese; thus, SO was not observed [9, 29].

The levels of physical activity (PASE) ranged from 0–312 points, with no significant difference between men and women. Motor function was generally high; 67 % of the individuals reached the maximum of 55 points in the M-MAS UAS-99 (range 39–55). This indicates that the stroke population in this study only had minor to moderate impairments and activity restrictions.

Next, we analysed potential denominators of mobility (SPPB). The co-variates were selected from measures of body composition, nutritional status, and physical activity (PASE and M-MAS). For the correlation and regression analyses between SPPB and MNA-SF, the “mobility” item was removed from the MNA-SF to avoid multicollinearity. For this analysis MNA-SF 11–12 points indicated normal nutritional status, whereas scores of ≤ 10 indicated risk for malnutrition, and this cut-off was used to dichotomise nutritional status. Considering all individuals, univariate analysis revealed significant associations between SPPB and age (r = −0.33, *P* = 0.044), PASE (r = 0.51, *P* < 0.001), nutritional status (MNA-SF without the “mobility” item) (r = 0.33, *P* < 0.001), and gender (r = 0.17, *P* = 0.054). Gender-separated associations are presented in Table 3. There were no univariate associations between SPPB and any body composition measure including FFMI in either the whole group or when separated by gender. The M-MAS showed a ceiling effect and was therefore not used in the multivariate regression analyses.

**Table 3:** Univariate associations between mobility (SPPB) and age, body composition, physical activity level and nutritional status, 1–3 years after stroke

	Women (*n* = 41)		Men (*n* = 93)	
Explanatory variables	r	*P*	r	*P*
Age, years	−0.29	0.065	−0.31	0.003
FFMI, (kg/m^2^)	0.015	0.89	−0.033	0.83
FMI, (kg/m^2^)	−0.012	0.94	−0.046	0.66
PASE (points, ≥0)	0.45	0.003	0.5	<0.001
MNA-SF	0.32	0.039	0.31	0.003

*Abbreviations*: *SPPB* Short Physical Performance Battery, Fat Mass, *FMI* Fat-Free Mass, *FFMI MNA-SF* Mini Nutritional Assessment - Short Form, *PASE* Physical Activity Scale for the Elderly

*P* < 0.05

We used the cut-off of ≤ 8 points to dichotomise mobility into high and low mobility [6] for logistic regression analyses. All estimates were adjusted for age (65–74 and 75–85 years) and gender. When the remaining variables were tested for multi-collinearity by cross-tabulation, the PASE scores (divided into tertiles indicating low, medium, and high physical activity), nutritional status, age (10-year intervals), and gender were selected as independent variables in the regression analyses with SPPB as the dependent variable. Table 4 shows the result of the univariate and the adjusted multivariate logistic regression analyses, indicating that low mobility was related to the risk of malnutrition (MNA-SF, OR 4.3, CI 1.7–10.5), low physical activity (PASE) (OR 6.5, CI 2.0–21.2), medium physical activity (PASE) (OR 3.5, CI 1.3–9.6) and, finally, every 10 years of increased age (OR 0.36, CI 0.15–0.85).

**Table 4:** Logistic regression with low mobility (SPPB) as dependent variable in 134 cases, 1–3 years after stroke

	Univariate model			Multivariate model		
	Odds ratio	95 % CI	*P*	Odds ratio	95 % CI	*P*
Explanatory variables						
PASE medium level	3.3	1.3 to 8.1	0.012	3.5	1.3 to 9.6	0.013
PASE low level	7.7	2.6 to 22.9	<0.001	6.5	2.0 to 21.2	0.02
MNA-SF (except mobility)^a^	4.3	1.9 to 9.8	<0.001	4.3	1.7 to 10.5	0.02
Gender	1.3	0.58 to 2.99	0.48	1.13	0.83 to 0.98	0.79
Age per 10 years	0.38	0.18 to 0.81	0.013	0.36	0.15 to 0.85	0.02

Analyses were adjusted for gender and age (per 10-year increment), R^2^ = Chi-square (χ^2^) = 13.8, *p* = 0.087 (Hosmer & Lemeshow)

*Abbreviations*: *MNA-SF* Mini Nutritional Assessment-Short Form, *PASE* Physical Activity

Scale for the Elderly (PASE; medium vs. high and low vs. high)

^a^ For this analysis MNA-SF was calculated with the mobility item omitted”

Interestingly, when the individual components of SPPB were tested for potential univariate associations, time for five chair-stands (s) revealed significant associations with physical activity (r = −0.35, *P* > 0.001), nutritional status (r = −0.22, *P* = 0.012), and FMI (r = 0.21, *P* = 0.017).

## Discussion

This study on ambulatory community-dwelling individuals one to 3 years after stroke displayed some confirmative and some novel findings. Not surprisingly, more than one in five individuals were obese. Reduced mobility (SPPB) and low gait speed in 29 % and 28 %, respectively, was also expected. The prevalence of sarcopenia was approximately 7 % according to the used definition. None of the individuals had SO. Fourteen percent of the individuals were identified as at risk for malnutrition, whereas none were classified as malnourished. The risk of malnutrition according to the MNA-SF, old age, and reduced levels of self-reported physical activity (PASE) were associated with an exponential risk for low mobility (SPPB). A somewhat unexpected result was that body composition measures (e.g., FMI and FFMI) were not related to SPPB scores. Therefore, the hypothesis that reduced FFMI was associated with reduced mobility could not be confirmed. However, the other hypotheses concerning the associations of the SPPB with nutritional status and self-reported physical activity were confirmed.

The prevalence of sarcopenia was unexpectedly low (7 %) in this cohort of post-stroke subjects and is generally in line with findings in community-dwelling older individuals [6]. The observed low prevalence is mainly related to the apparently well-preserved muscle mass of the subjects, as they appeared to have an even greater muscle mass than the Swiss healthy reference population. This finding is difficult to explain, but it may relate to the fact that the weight was higher in the post-stroke cohort and that obesity was common, which is known to be related to higher muscle mass [31, 32]. It is also important to realise that most of the included individuals in this study had suffered a minor stroke without a pronounced hemiparesis and with preserved walking capacity. In Sweden, 74 % of stroke patients are released to their home after acute stroke. This fact indicates that the current study is fairly representative of community-dwelling post-stroke individuals. There is a stroke-specific type of sarcopenia [31, 32] that primarily occurs in patients with more severe disease than observed among these study participants. To our knowledge, this is the first report on the prevalence of sarcopenia in community-dwelling individuals after stroke.

The patients’ diet histories were not recorded; thus, it is difficult to establish whether a high energy intake or physical inactivity was the major cause for obesity. Despite inconsistent findings, whole-body FM appears to increase between 6 and 12 months post-stroke, whereas no change in FFM has been reported over time [33, 34]. Interestingly, recent studies have reported lower mortality, improved functional outcomes, and a lower risk for re-admission from recurrent stroke in obese compared with lean stroke patients [31, 35]. This is sometimes referred to as the obesity paradox [35].

The prevalence of malnutrition or being at risk for malnutrition (14 %) according to MNA-SF in the present study was lower than the corresponding 37 % reported from a European compilation of community-living older adults (age 79 years) without stroke [36]. Although the mean age was 5 years lower in the current study group of post-stroke community-living individuals, the combined findings indicate that the study group was in fairly good condition.

As illustrated by the M-MAS UAS-99 results, motor function was high. If more disabled individuals had been included, the prevalence rates of sarcopenia and SO would likely have been higher. The observation of overweight and obesity not being associated with reduced mobility was interesting and somewhat unexpected and contrasted with earlier findings [9]. As already indicated, it was also unexpected that alterations in FFMI were not related to mobility as measured by the SPPB. This result suggests that muscle mass is not always crucial for function, which has also been observed in other studies performed in elderly populations [32, 37]. Perhaps this lack of relationship between muscle mass and function is even more pronounced in stroke survivors for whom neurological damage also contributes to strength and function.

The reduced PASE scores recorded in the current study are in agreement with previous reports of community-living individuals with stroke, corresponding to 67 % of gender- and age-matched control values [38]. It is unlikely that the observed reduced mobility and physical activity were primarily related to neurological damage due to the previous stroke because only a few individuals had motor impairments.

The current study has limitations that need to be acknowledged. About half of the eligible population could not be examined, which may limit the generalisability of the results. Individuals with more severe disabilities (e.g., unable to stand safely) could not perform the BIA procedure, which affects the generalisation of the results to more disabled stroke patients, e.g., nursing-home residents. Individuals in the Swiss reference population conducted BIA in the supine position. In the current study BIA was performed in standing position, which may differ from BIA measurement performed in lying. Furthermore, individuals with subarachnoid haemorrhage were not included. All individuals in this study were community-dwelling, indicating better functional and nutritional status compared with nursing home residents. According to the International Classification of Functioning Disability and Health (ICF), disability is an umbrella term for impairments, activity limitations, and participation restrictions [39]. The measures of this study covered a great part of the ICF description of disability by including the motor function (M-MAS UAS-99), mobility (SPPB), and gait speed (10 mWT). Activity limitations and to some extent also participation restrictions were covered by the PASE, measuring exercise, leisure-time activities and voluntary work. Moreover, the cross-sectional design of the study limits the possibility to make causal inferences. Further studies on body composition and sarcopenia in stroke populations with minor to moderate stroke are needed [34].

## Conclusions

We conclude that in community-living subjects assessed one to 3 years after stroke, reduced mobility according to SPPB was related to risks of malnutrition (according to the MNA-SF), low and medium physical activity (according to PASE) but not to alterations in body composition, e.g., FFMI and FMI. Obesity, sarcopenia, and risk of malnutrition were observed in about one-third of the study population. These results suggest that stroke rehabilitation efforts could benefit from promoting a healthy and nutritious diet and physical activity to optimise mobility, reduce obesity, and avoid sarcopenia. Intervention studies are needed to address the potential benefits of such lifestyle changes.

## Abbreviations

BIA: bio-impedance analysis
BMI: body mass index
CCI: Charlson Co-morbidity index
CRP: c-reactive protein
FFM: fat-free mass
FFMI: fat-free mass index
FM: fat mass
FMI: fat mass index
HDL: high-density lipoprotein
IGF-1: insulin-like growth factor-1
LDL: low density lipoprotein
M-MAS UAS-99: modified motor assessment scale uppsala university hospital-99
MNA-SF: the mini nutritional assessment-short form
OR: odds ratio
PASE: physical activity scale for the elderly
SO: sarcopenic obesity
SPMSQ: short portable mental status questionnaire
SPPB: short physical performance battery
TIA: transient ischaemic attack

## References

[1] Gariballa SE (2000). Nutritional factors in stroke. Br J Nutr.

[2] Foley NC, Salter KL, Robertson J, Teasell RW, Woodbury MG (2009). Which reported estimate of the prevalence of malnutrition after stroke is valid?. Stroke.

[3] Jönsson AC, Lindgren I, Norrving B, Lindgren A (2008). Weight loss after stroke: a population-based study from the Lund Stroke Register. Stroke.

[4] Aquilani R, Scocchi M, Iadarola P, Franciscone P, Verri M, Boschi F, Pasini E, Viglio S (2008). Protein supplementation may enhance the spontaneous recovery of neurological alterations in patients with ischaemic stroke. Clin Rehabil.

[5] Fielding RA, Vellas B, Evans WJ, Bhasin S, Morley JE, Newman AB, van Kan Abellan G, Andrieu S, Bauer J, Breuille D (2011). Sarcopenia: an undiagnosed condition in older adults. Current consensus definition: prevalence, etiology, and consequences. International working group on sarcopenia. J Am Med Dir Assoc.

[6] Cruz-Jentoft AJ, Baeyens JP, Bauer JM, Boirie Y, Cederholm T, Landi F, Martin FC, Michel JP, Rolland Y, Schneider SM (2010). Sarcopenia: European consensus on definition and diagnosis: report of the European working group on sarcopenia in older people. Age Ageing.

[7] Jørgensen L, Jacobsen BK (2001). Changes in muscle mass, fat mass, and bone mineral content in the legs after stroke: a 1 year prospective study. Bone.

[8] Cruz-Jentoft AJ, Landi F, Schneider SM, Zúñiga C, Arai H, Boirie Y, Chen LK, Fielding RA, Martin FC, Michel JP (2014). Prevalence of and interventions for sarcopenia in ageing adults: a systematic review. Report of the International Sarcopenia Initiative (EWGSOP and IWGS). Age Ageing.

[9] Zamboni M, Mazzali G, Fantin F, Rossi A, Di Francesco V (2008). Sarcopenic obesity: a new category of obesity in the elderly. Nutr Metab Cardiovasc Dis.

[10] Prado CM, Wells JC, Smith SR, Stephan BC, Siervo M (2012). Sarcopenic obesity: A Critical appraisal of the current evidence. Clin Nutr.

[11] Batsis JA, Mackenzie TA, Barre LK, Lopez-Jimenez F, Bartels SJ (2014). Sarcopenia, sarcopenic obesity and mortality in older adults: results from the National Health and Nutrition Examination Survey III. Eur J Clin Nutr.

[12] Charlson ME, Pompei P, Ales K, MacKenzie CR (1987). A new method of classifying prognostic comorbidity in longitudinal studies; developement and validation. J Chronic Dis.

[13] Olsson T, Terent A, Lind L (2005). Charlson Comorbidity Index can add prognostic information to Rapid Emergency Medicine Score as a predictor of long-term mortality. Eur J Emerg Med.

[14] Pfeiffer E (1975). A short portable mental status questionnaire for the assessment of organic brain deficit in elderly patients. J Am Geriatr Soc.

[15] Roccaforte WH, Burke WJ, Bayer BL, Wengel SP (1994). Reliability and validity of short portable mentale status questionnaire administered by telephone. J Geriatr Psychiatry Neurol.

[16] Guralnik JM, Simonsick EM, Ferrucci L, Glynn RJ, Berkman LF, Blazer DG, Scherr PA, Wallace RB (1994). A short physical performance battery assessing lower extremity function: association with self-reported disability and prediction of mortality and nursing home admission. J Gerontol.

[17] Tyson S, Connell L (2009). The psychometric properties and clinical utility of measures of walking and mobility in neurological conditions: a systematic review. Clin Rehabil.

[18] Guralnik JM, Ferrucci L, Pieper CF, Leveille SG, Markides KS, Ostir GV, Studenski S, Berkman LF, Wallace RB (2000). Lower extremity function and subsequent disability: consistency across studies, predictive models, and value of gait speed alone compared with the short physical performance battery. J Gerontol A Biol Sci Med Sci.

[19] Carr JH, Shepherd RB, Nordholm L, Lynne D (1985). Investigation of a new motor assessment scale for stroke patients. Phys Ther.

[20] Vahlberg B, Cederholm T, Lindmark B, Zetterberg L, Hellström K (2013). Factors Related to Performance-Based Mobility and Self-reported Physical Activity in Individuals 1–3 Years after Stroke: A Cross-sectional Cohort Study. J Stroke Cerebrovasc Dis.

[21] Ostir GV, Volpato S, Fried LP, Chaves P, Guralnik JM, Study WsHaA (2002). Reliability and sensitivity to change assessed for a summary measure of lower body function: results from the Women’s Health and Aging Study. J Clin Epidemiol.

[22] Washburn RA, Smith KW, Jette AM, Janney CA (1993). The Physical Activity Scale for the Elderly (PASE): development and evaluation. J Clin Epidemiol.

[23] Washburn RA, McAuley E, Katula J, Mihalko SL, Boileau RA (1999). The Physical Activity Scale for the Elderly (PASE): evidence for validity. J Clin Epidemiol.

[24] Lindahl MHL, Petersen A, Truelsen T, Boysen G (2008). Self-reported physical activity after ischemic stroke correlates with physical capacity. Adv Physiother.

[25] Bauer JM, Kaiser MJ, Anthony P, Guigoz Y, Sieber CC (2008). The Mini Nutritional Assessment--its history, today’s practice, and future perspectives. Nutr Clin Pract.

[26] Kaiser MJ, Bauer JM, Ramsch C, Uter W, Guigoz Y, Cederholm T, Thomas DR, Anthony P, Charlton KE, Maggio M (2009). Validation of the Mini Nutritional Assessment short-form (MNA-SF): a practical tool for identification of nutritional status. J Nutr Health Aging.

[27] Schutz Y, Kyle UU, Pichard C (2002). Fat-free mass index and fat mass index percentiles in Caucasians aged 18–98 y. Int J Obes Relat Metab Disord.

[28] Abernathy RP, Black DR (1996). Healthy body weights: an alternative perspective. Am J Clin Nutr.

[29] Engvall IL, Elkan AC, Tengstrand B, Cederholm T, Brismar K, Hafstrom I (2008). Cachexia in rheumatoid arthritis is associated with inflammatory activity, physical disability, and low bioavailable insulin-like growth factor. Scand J Rheumatol.

[30] Pietrobelli A, Rubiano F, St-Onge MP, Heymsfield SB (2004). New bioimpedance analysis system: improved phenotyping with whole-body analysis. Eur J Clin Nutr.

[31] Doehner W, Schenkel J, Anker SD, Springer J, Audebert HJ (2013). Overweight and obesity are associated with improved survival, functional outcome, and stroke recurrence after acute stroke or transient ischaemic attack: observations from the TEMPiS trial. Eur Heart J.

[32] Legrand D, Vaes B, Matheï C, Swine C, Degryse JM (2013). The prevalence of sarcopenia in very old individuals according to the European consensus definition: insights from the BELFRAIL study. Age Ageing.

[33] English C, Thoirs K, Coates A, Ryan A, Bernhardt J (2012). Changes in fat mass in stroke survivors: a systematic review. Int J Stroke.

[34] English C, McLennan H, Thoirs K, Coates A, Bernhardt J (2010). Loss of skeletal muscle mass after stroke: a systematic review. Int J Stroke.

[35] Andersen KK, Olsen TS (2013). The obesity paradox in stroke: Lower mortality and lower risk of readmission for recurrent stroke in obese stroke patients. Int J Stroke.

[36] Kaiser MJ, Bauer JM, Rämsch C, Uter W, Guigoz Y, Cederholm T, Thomas DR, Anthony PS, Charlton KE, Maggio M (2010). Frequency of malnutrition in older adults: a multinational perspective using the mini nutritional assessment. J Am Geriatr Soc.

[37] Schaap LA, Koster A, Visser M (2012). Adiposity, muscle mass, and muscle strength in relation to functional decline in older persons. Epidemiol Rev.

[38] Danielsson A, Meirelles C, Willen C, Sunnerhagen KS (2013). Physical activity in community-dwelling stroke survivors and a healthy population Is not explained by motor function only. PM R.

[39] World Health Organization (2008). International classification of functioning, disability and health: ICF.

